# Endovascular treatment of an intraosseous arteriovenous malformation of the mandible in a child. A case Report

**DOI:** 10.4317/jced.54550

**Published:** 2018-02-01

**Authors:** Paolo Cariati, Ana-Belén Marín-Fernández, Miguel-Ángel Julia-Martínez, Miguel Pérez-de Perceval-Tara, Darío Sánchez-López, Ildefonso Martínez-Lara

**Affiliations:** 1 Maxillofacial Surgery Resident. Oral and Maxillofacial Surgery Department, Hospital Virgen de las Nieves, Granada, Spainn; 2 Maxillofacial Surgeon. Oral and Maxillofacial Surgery Department, Hospital Virgen de las Nieves, Granada, Spain

## Abstract

The modern application of interventional radiology techniques is revolutionizing the treatment of the vascular malformations of the head and neck. In fact, the morbidity related with the surgical extirpation of the malformation might be drastically reduced with the use of an endovascular approach. The present report describes the case of a 11 years old male affected by a large intraosseous arteriovenous malformation of the mandible. The coil embolization of the main drainage vein caused the spontaneous regression of the lesion and avoided a mutilating surgery and severe psychological sequels. A multidisciplinary approach of these case is mandatory. A careful clinical and radiological study of the patient is essential for a proper management. The choice of the treatment should be based on the location and extension of the malformation, age of the patient, experience with endovascular techniques and clinic.

** Key words:**Endovacualr approach, intraosseous arteriovenous malformation, head and neck, child.

## Introduction

Arteriovenous malformations (AVMs) are uncommon and congenital vascular malformations representing only 1.5% of all vascular anomalies. However, 50% of these affect the oral and maxillofacial region. The lip and the oral mucosa are the most frequently affected areas (1).

In 1996, the International Society for the Study of Vascular Anomalies (ISSVA) adopted and reviewed the classification created by Mulliken and Glowacki in 1982. They proposed the division of these anomalies in two groups: 1) tumors and 2) malformations. The vascular malformations could be divided into low-flow (capillary, venous or lymphatic) and fast flow malformations (arterial malformation and arteriovenous fistula) (2,3). Although these lesions are congenital, they usually grow during adolescence and adulthood. Unfortunately, the spontaneous regression is extremely rare. Pain, tissue expansion, ulceration and bleeding may appear when the malformation reaches a significant size (4). Treatment constitute a challenge, and a multidisciplinary approach is strongly recommended. Endovascular techniques are currently considered the treatment of choice, and are often combined with the surgical resection of the lesion. However, in many cases, treatment is only palliative (5,6). The main aim of the present report is to show the brilliant results obtained with radiologically assisted embolization in the treatment of a large high-flow intraosseous arteriovenous malformation.

## Case Report

**Figure 1:**
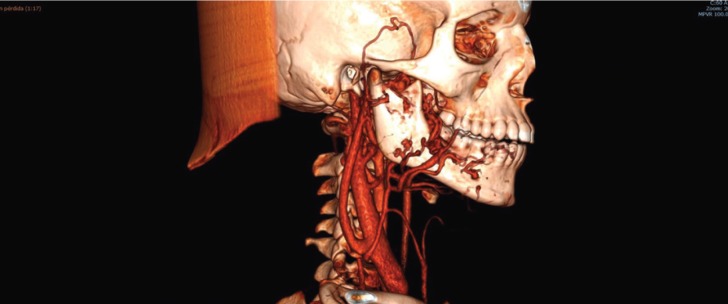
3D CT image of the arteriovenous malformation extending from the right mandibular body to the homolateral pterygoid region and infratemporal fossa.

**Figure 2:**
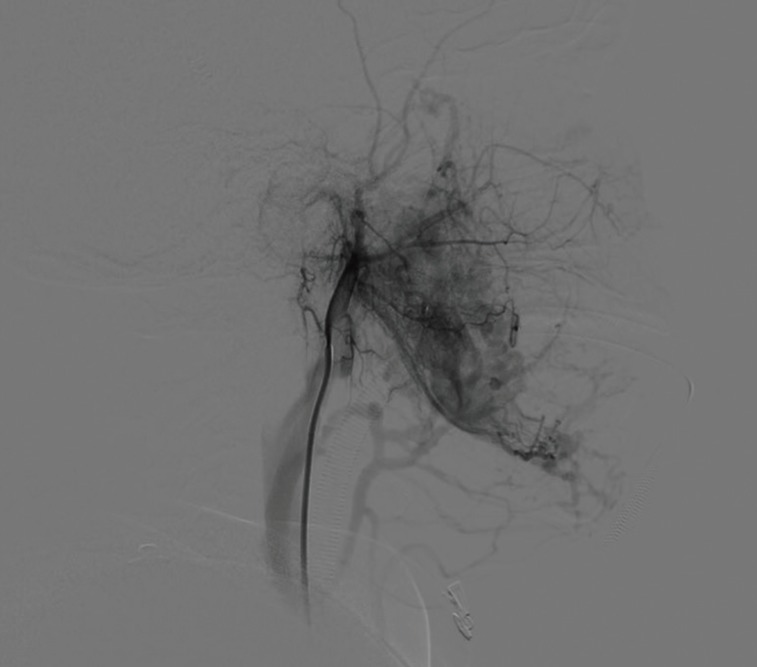
Arteriographic image of the malformation. The picture shows the great volume of the lesion and the big drainage vein used for treating the malformation.

**Figure 3:**
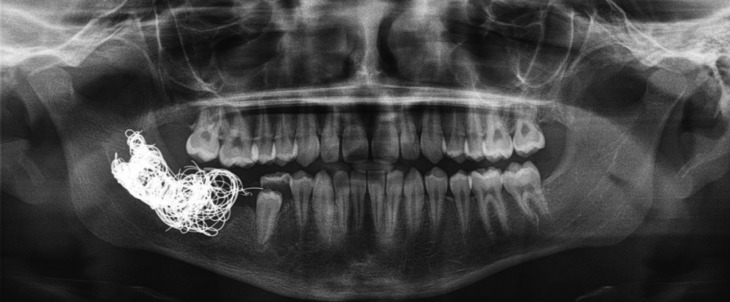
OPG image 3 month after treatment. The image shows the presence of the material used for the vein embolization.

A 11 years old male was referred to the emergency department of Virgen de las Nieves University Hospital due to a slight periodontal bleeding of 5 days of evolution. An orthopantomography was performed to focus the diagnosis. The OPG evidenced a dubious periapical image compatible with a small cyst of dental origin. Hence, the doctors of the emergency service decided to contact with the maxillofacial surgery department. A new clinical examination evidenced a spontaneous alveolar bleeding and high mobility of the first and second molars of the fourth mandibular quadrant. The patient denied cervicofacial trauma or pathologies of the coagulation system. Thus, a CT scan of the cervicofacial area was performed to reach a diagnosis. Surprisingly, this test evidenced a large high-flow intraosseous arteriovenous malformation extending from the right mandibular body to the homolateral pterygoid region and the infratemporal fossa (Fig. 1). Moreover, a selective arteriography and a MRI of head and neck were also carried out to identify the limits of the malformation and the key vessel (Fig. 2). Considering these findings, we contacted with the interventional radiology unit of the Hospital in order to plan a combined approach. However, after a careful analysis we decided to treat the patient only with an endovascular approach. Specifically, a very large drainage vein was identified with the radiological tests. Hence, we hypothesized that the embolization of this vein and the selective embolization of the main arterial vessels might be enough to treat the pathology. Thus, under general anesthesia and femoral approach, the embolization of the main drainage vein and of the most accessible arteries was carried out. The patient was maintained intubated and sedated for 24 hours and he was extubated when the bleeding had completely stopped. He was discharged from the hospital 7 days after the procedure. In fact, a control arteriography performed 5 day after embolization showed a significant reduction of the size of the lesion. A MRI performed three months after the procedure evidenced the complete regression of the lesion. Furthermore, in a new control 18 month after the patient has no clinical signs of recurrence (Fig. 3). Hence, this approach could avoid an extremely mutilating surgery and severe psychological sequels.

## Discussion

Arteriovenous malformations can be extremely serious and dangerous in some cases. The modern application of interventional radiology techniques is revolutionizing the treatment of these lesions. Specifically, the morbidity related with the surgical extirpation might be greatly reduced with the use of these procedures (6). In this paper we present the case of a 11-year-old male affected by a wide intraosseous arteriovenous malformation of the mandible. The main characteristics of the case is the choice to treat the injure through the embolization of the major drainage vein. In fact, most of the arteriovenous malformations hound in the head and neck region are usually treated with the selective embolization of the main arteries (7,8). Moreover, the isolated embolization of the main drainage vein might be dangerous with intracranial arteriovenous malformations (9). In our case, the abundant collateral circulation generated by the malformation hindered the selective arterial embolization. The collateral circulation and de extension of the injure also hampered a surgery with clean margins. Fortunately, the embolization of the main drainage vein caused the spontaneous regression of the malformation and avoided an extremely mutilating surgery. In our opinion, a multidisciplinary approach of these case is mandatory. A careful clinical and radiological study of the patient is essential for a proper management. The choice of the treatment should be based on location and extension of the malformation, age of the patient, clinic and experience with endovascular techniques. For instance, surgery is more suit-able in cases where the lesion is well defined and far from important anatomical structures. According with several papers, the combination of embolization and surgery is the optimal choice of treatment (7). In our opinion, endovascular techniques represent an important therapeutic weapon for the management of arteriovenous malformations of the head and neck. They should be always considered as the first therapeutic step in symptomatic arteriovenous malformations of the cervicofacial area.
